# Compassion as a tool for allyship and anti-racism

**DOI:** 10.3389/fpsyg.2023.1143384

**Published:** 2023-04-11

**Authors:** Melissa M. Karnaze, Ramya M. Rajagopalan, Lisa T. Eyler, Cinnamon S. Bloss

**Affiliations:** ^1^The Herbert Wertheim School of Public Health and Human Longevity Science, University of California, San Diego, La Jolla, CA, United States; ^2^Center for Empathy and Technology, T. Denny Sanford Institute for Empathy and Compassion, University of California, San Diego, La Jolla, CA, United States; ^3^Department of Psychiatry, School of Medicine, University of California, San Diego, La Jolla, CA, United States; ^4^Center for Empathy and Compassion Training in Medical Education, T. Denny Sanford Institute for Empathy and Compassion, University of California, San Diego, La Jolla, CA, United States

**Keywords:** compassion, empathy, allyship, anti-racism, systemic racism

## Abstract

Racist systems, policies, and institutions subvert the quality of life for minoritized individuals and groups, across all indicators, from education and employment, to health, to community safety. Reforms to address systemic racism may be accelerated with greater support from allies who identify with the dominant groups that derive advantage from such systems. Although enhancing empathy and compassion for impacted individuals and groups may foster greater allyship with and support of minoritized communities, little work to date has assessed the relationships among compassion, empathy, and allyship. After reviewing current work in the area, this perspective offers insights into the utility and specific components of a compassion-based framework that can be used to combat racism, using findings from a survey study in which we investigated the relationship between validated psychometric measures of compassion and allyship with minoritized communities. Several subdomains of compassion, as measured among individuals identifying as non-Black, correlate significantly with levels of felt allyship with Black or African American communities. These findings inform recommendations for compassion-focused research, including development and testing of interventions to promote allyship, advocacy, and solidarity with minoritized groups, and support efforts to undo longstanding structural racisms that have patterned inequality in the United States.

## Introduction

The SARS-CoV-2 pandemic and recent fatal encounters with law enforcement have disproportionately impacted communities of color in the United States., highlighting four centuries of devastating structural racism as the bedrock of persistent racial disparities in health and policing. Those who have historically benefitted from racist or discriminatory systems and practices are increasingly being called on to recognize systemic racism and to act in allyship or solidarity with minoritized communities to address inequities and reform oppressive systems.

One potential tool for developing allyship is cultivating compassion. Compassion includes recognizing suffering, and feeling positive regard for distressed persons, which motivates helping behavior. Dispositional compassion is associated with stronger social relationships; in addition, people can be prompted to act more kindly toward others even when others are viewed as being in opposition to one’s own group (Klimecki, 2019). The role of compassion training in promoting stronger allyship remains a nascent area of inquiry, especially in relation to high-impact advocacy work and actions to promote social justice, which the United Nations has broadly defined as “fair and compassionate distribution of the fruits of economic growth” (Department of Economic and Social Affairs–DESA/UN, 2006). Misconceptions about the nature of compassion (e.g., that it is equivalent to pity) may make it seem antithetical to true solidarity with those who are oppressed. Existing work in this area suggests that a central component of social justice action is feeling for the suffering of others as a motivator of efforts to help alleviate that suffering (Viray and Nash, 2014). In this way, social justice efforts can be viewed as embodying compassionate acts aimed at supporting or advocating for those who suffer from social inequities, and “allies” include members of a dominant social group not directly experiencing suffering from such inequities but who perform acts of compassion based on their empathy for those who are (LeBlanc et al., 2022).

It is important to differentiate allyship from the notion of saviorism. Allies perform roles supportive to members of non-dominant social groups, rather than monopolizing leadership roles, and their status of allyship is defined by the groups they purport to help (Smith et al., 2016). Saviorism, in contrast, refers to actions that claim to help people in minoritized groups, but a closer examination reveals that these actions are self-serving rather than beneficial for the community “in need” of support. Examples might include engaging in charity work to boost one’s reputation or social standing, or to resolve guilt associated with acknowledging privilege traditionally afforded to one’s dominant social group. Such actions can be viewed as exploiting the plight of minoritized communities for self-serving aims and can have negative long-term consequences for these communities (e.g., Anny, 2021; Williams et al., 2021). Racial justice allyship can be rewarding, but it must involve the “deliberate dismantling of explicit, implicit, and systemic patterns of injustice” which requires courage to confront the status quo and face any negative consequences from one’s social group in doing so (Williams et al., 2023). Courage can take time to develop, and Williams and colleagues have outlined several practical exercises based on cognitive-behavioral approaches to guide action even when feeling anxious or afraid of potentially adverse responses from members of socially dominant groups.

Rectifying racist policies will require allyship from those who currently benefit from such policies. Unfortunately, even when people state their intentions to be allies, self-reports fall short of actions as assessed in low-stakes scenarios in the laboratory setting (Williams et al., 2021), highlighting the shortage of white allies and the need for effective methods to cultivate more allies. Compassion is uniquely situated to be compatible with and to enhance allyship. First, there is evidence that positive feelings inspired by an “outgroup,” and feeling motivated to interact with members of an “outgroup,” are associated with allyship (Pittinsky et al., 2011; Williams and Sharif, 2021). Similarly, compassion should also motivate prosocial behavior toward those suffering from discrimination. Second, feelings of compassion, or at minimum a compassionate mindset, should be conducive to an ally’s social interactions with members of dominantly situated social groups, when the goal is to confront injustices in order to change the status quo. More research is needed to understand how compassion and empathy relate to feelings of allyship in support of minoritized communities. We present preliminary work from a field survey in which we sought to assess the relationship between compassion and allyship with Black or African American communities among people who do not identify as Black. We also discuss how the findings point to possible areas for further research to promote a compassion framework for allyship, advocacy, and solidarity.

## Authors’ positionality statement

Our team of co-authors hold positionalities that in combination have shaped our collective approach to this research. All authors identify as cis-gender female, and our lived experiences within our racial/ethnic identities (Asian, mixed-race, South Asian, and White), as well as our research interests, have together informed our perspectives on the intersection of compassion and social justice. Our scholarship encompasses a diverse set of expertises in emotion and emotion regulation, beliefs about emotion, public health, racial health disparities and health equity, ethics, precision medicine, intervention research, and medical education. This study and our findings were shaped by our collective research expertise, lived experiences, and participation in anti-racism and compassion cultivation training conducted at our institution in 2020.

## Compassion and allyship with Black and African American people

In June 2020, as part of a larger study of compassion and empathy during the pandemic (Karnaze et al., 2022), we fielded a survey to U.S.-based adults recruited from two convenience samples, Amazon Mechanical Turk and Qualtrics Online Panels. For the present analysis, we focused on the 861 individuals surveyed who did not self-identify as Black or African American. In this subsample, about half identified as female (*N* = 402). In terms of race/ethnicity, a majority identified as White (*N* = 766) and the rest identified as Asian, American Indian or Alaska Native, Pacific Islander, mixed race, or other race (*N* = 95), with three-quarters reporting ethnicity as non-Hispanic/Latino (*N* = 642; versus Hispanic/Latino).

Using psychometrically validated measures, we examined partial correlations between a measure of felt allyship with “Black people” (Neufeld et al., 2019) and: (a) dispositional compassion (Hwang et al., 2008), (b) dispositional empathy (Spreng et al., 2009), and (c) five components of compassion as informed by Eastern contemplative practices and contemporary emotion theory (Strauss et al., 2016; Gu et al., 2020). These correlations controlled for binary sex/gender, social desirability, and political ideology, as partisan interests in the U.S. may shape perceptions about which social groups have “valid” emotions of distress and suffering and thus which are deserving of compassion (Shields, 2005).

**Figure 1:**
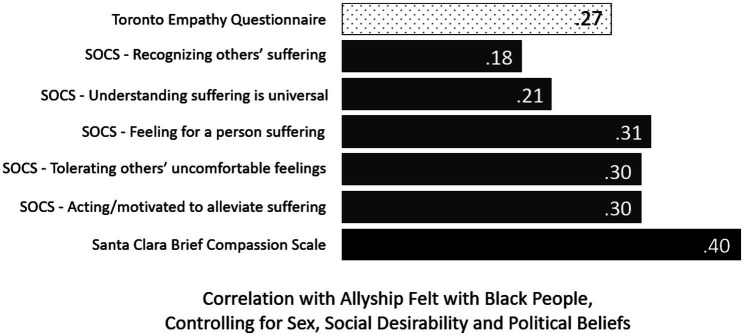
Partial correlations of empathy and compassion with felt allyship with Black People, controlling for sex, social desirability, and political beliefs. SOCS is the abbreviation for the Sussex Oxford Compassion for Others Scale and is used to refer to each of the five subscales of the measure.

Dispositional empathy and compassion scores were all correlated with allyship (Figure 1), although the correlation of allyship with compassion was statistically significantly stronger than with empathy. Of the compassion subdomains, three showed moderately strong relationships with allyship. Feeling for a person suffering and emotionally connecting with their distress showed the strongest correlation. Two other components had moderately strong correlations with allyship, tolerance of uncomfortable feelings elicited in response to the suffering of another person, and acting or feeling motivated to act to alleviate another person’s suffering.

Next, we discuss our preliminary findings in the context of these results and draw upon existing research to suggest areas for further investigation on the utility of cultivating compassion to increase allyship and solidarity with minoritized individuals. Given that applying compassion in a social justice context must contend with several challenges, we also discuss elements to avoid in designing compassion-based interventions.

## Areas for future research on compassion to promote allyship

Our research recommendations are organized by the five elements of compassion as defined by Strauss and colleagues, which we assessed in our survey (Strauss et al., 2016; Gu et al., 2020). Where appropriate, we discuss the progression of these elements in more specificity as outlined by Stevens and Taber’s (2021) conceptual model of how empathy and compassion translate to prosocial behavior.

### Recognition of suffering

Theories of compassion define the recognition of distress or suffering experienced by another person as the first step in being able to compassionately respond to their suffering. Our preliminary work revealed a small correlation between recognition of suffering in general terms (e.g., “I notice when others are feeling distressed”) and allyship. To increase potential allies’ recognition of suffering *experienced by marginalized groups*, research should test whether strengthening the capacities of those who have not experienced discrimination leads them to understand or take the perspective of those who have.

When adversity based on discrimination is not experienced directly, there are outlets for escaping or avoiding recognition of its detrimental impacts, a luxury to which marginalized groups do not have access (Sadavoy and Zube, 2021). Enhancing skills for perspective-taking can enhance recognition of and action against social injustice, while its absence can aggravate social conflict, prejudice, and discrimination (Garcia et al., 2021). Research shows that perspective-taking promotes compassion cultivation (Klimecki, 2019), reduces stereotyping and prejudice against marginalized groups (Todd et al., 2011; Matsuda et al., 2020), and increases the likelihood of engaging in helping actions toward outgroups (Batson et al., 2002).

In addition, further studies are needed to determine whether focusing attention on the effects of racist practices and policies increases recognition of suffering and felt allyship with individuals who experience the brunt of these policies. This may help reduce any fundamental attribution error when members of dominant groups encounter the suffering of minoritized individuals; the fundamental attribution error involves blaming individual attributes or choices for adverse outcomes—rather than structural or systemic factors—and can result in assigning blame to the individual suffering from discriminatory practices or policies, rather than systems that perpetuate discrimination. Focusing on racist systems rather than individual “bad actors” who perpetrate racist acts may also help reduce any cognitive dissonance that members of dominant groups might otherwise resolve by, for instance, viewing themselves more favorably than “racists,” which shifts focus away from one’s potential for agency and allyship with minoritized groups. Experiments are needed to test whether reducing these tendencies to focus on individuals who commit racist acts (rather than focusing on racist systems) increases recognition of suffering due to racism and results in felt empathy and compassion.

### Feeling for persons in distress

After recognition of suffering occurs, the next step in compassionate responding is to share the feelings of the person in distress, which is referred to as empathy. In our preliminary work, feeling for a person suffering and emotionally connecting with their distress was the component of compassion that had the strongest correlation with allyship. We propose that interventions for enhancing allyship among individuals and groups with traditionally racially privileged identities should emphasize minoritized individuals’ *personal experiences* of suffering resulting from racism, rather than raising awareness about discrimination in more abstract terms. For example, trainings that center personal narratives or vignettes humanizing those impacted by institutional racism and highlighting experiences of suffering in the context of lived experiences, may offer functional levers for increasing allyship with marginalized communities.

Feeling for a person suffering and emotionally connecting with their distress can involve both down-regulating feelings that might interfere with empathy and up-regulating positive feelings and positive regard for the person in distress. In future work, perspective-taking and other evidence-based affect regulation strategies could be employed to up-regulate positive “feelings for” others including care and concern for individuals of minoritized groups.

### Tolerance of unpleasant feelings

Empathizing with others’ suffering can involve discomfort, so having the ability to tolerate unpleasant feelings with the goal of maintaining empathy is important for then shifting to feelings of compassion and a desire to help the person in distress. We found that allyship was correlated with tolerance of uncomfortable feelings elicited in response to the suffering of another person (e.g., fear, disgust, distress) in order to remain accepting of and open to the person suffering. Experimental research shows that compassion and empathy for others can be modulated (Leiberg et al., 2011), as can acceptance of unpleasant emotions (Wolgast et al., 2011), a necessary condition for having compassion when witnessing another’s suffering (Stevens and Taber, 2021). To increase such tolerance, researchers can test the efficacy of interventions designed to facilitate recognition and acceptance of one’s own reactions to others’ suffering, for example through mindfulness practice (Magee, 2021), especially in contexts that trigger motivations to down-regulate unpleasant feelings so as to experience less guilt or reactivity about one’s complicity in racist actions or systems (Ford et al., 2022).

Relatedly, one way that *intolerance* of unpleasant feelings can manifest is through pity, or well-intentioned “helping” acts, such as those exemplary of “White saviorism.” These actions tend to otherize marginalized groups and center the doer’s perspectives and desires to feel good over the emotions and long-term wellbeing of those in need of allyship (Anny, 2021). Thus, it is vital that future research efforts continue to clearly distinguish compassion from pity, which may require testing the efficacy of interventions that foster and sustain emotional connection with those suffering to mitigate the tendency to self-focus and otherize.

### Viewing suffering as a common human experience

Strauss et al. (2016) included understanding of the universality of human suffering as an important component of compassion, as this mindset has been part of Eastern contemplative practices for fostering compassion. Being able to acknowledge another’s suffering as normative to human experience should minimize negative judgment of an individual’s suffering, which can otherwise prevent compassion. Our preliminary work revealed a small correlation between allyship and viewing suffering as universal or adopting a “lovingkindness” orientation to all individuals. However, it can be challenging to translate this element of compassion, understanding the universality of human suffering, into allyship with specific groups. It is important to acknowledge unpleasant feelings and implicit biases that can arise when acknowledging the existence of *differential* harms and suffering among different social groups, in order to enhance feelings of allyship with members of those groups who experience disproportionate harms. Leveraging compassion to enhance allyship with minoritized groups should present these components in a nuanced way and minimize unintentional consequences. For example, viewing suffering as universal should not be engaged in at the expense of gaining a better understanding of the unique experiences of each individual, or of the experiences of suffering that may be more common to one minoritized community relative to another. One way to discuss universality of human suffering could be to include concrete examples of how racist systems harm not just minoritized communities but also individuals and groups with privileged identities in society (McGhee, 2022).

More broadly, interventions should ensure that encouraging a lovingkindness orientation does not result in harmful coping or defense mechanisms such as spiritual bypassing to avoid dealing with uncomfortable feelings (Fox et al., 2017). Nor should they result in complicit attitudes toward proponents of racist systems, defended by beliefs that “everyone suffers” or “everyone is just doing their best.” The view of suffering as universal should instead be harnessed to frame a more equitable and less racist society as a means to improving quality of life and reducing suffering for *all* groups, including those who passively benefit from oppressive systems.

### Acting and feeling motivated to help persons in distress

The final steps of compassionate responding include feeling compassion for another in distress, which prompts motivation to help them, and taking action to help. In our preliminary work, self-reports of acting or feeling motivated to act to alleviate another person’s suffering had a moderately strong correlation with allyship. Interventions for promoting a compassionate desire to act could raise awareness of specific policies and practices that disproportionately lead to both immediate and longer-term harm or suffering among minoritized individuals and groups, while also framing these policies and practices as modifiable and amendable.

Existing research suggests that compassion, unlike empathy, should be resistant to burnout (Klimecki et al., 2014; Stevens and Taber, 2021). It is important to prioritize research on how individuals engaged in political activism maintain positive feelings of compassion for those suffering from oppression without experiencing empathy burnout and disengaging. For instance, Stevens and Taber (2021) propose that to engage in prosocial behavior rather than empathic distress, one must successfully self-regulate unpleasant feelings that are part of empathy when encountering another’s suffering. Acceptance of one’s unpleasant feelings (Hayes et al., 1996) and self-compassionate responding to one’s unpleasant feelings (Neff, 2003) may help in the maintenance of self-regulation and sustained compassion.

While we found that acting or feeling motivated to act to alleviate another person’s suffering was related to reports of allyship, future research must test whether increasing such feelings results in actual action taken in solidarity with individual members of minoritized groups. For example, future research could leverage the broaden-and-build theory of positive emotion (Huppert et al., 2004), and test whether compassion and other prosocial emotions can promote divergent systems-level thinking. Such thinking can generate innovative action steps to advance the reform of structures and policies that are racist or lead to inequitable outcomes.

Given that allyship involves action to support minoritized communities, through individual behavioral changes and through structural changes that lead to more equitable social practices, systems, and institutions, it is important to track the long-term outcomes of compassion-based actions in support of enhancing allyship. It is equally important to define and track any unintended consequences of actions that are motivated by compassion; while feelings of compassion and actions taken to help others who are suffering can have good intentions, the implications of those actions need to be evaluated by those who are experiencing suffering or discrimination. Similarly, actions motivated by compassion should result in allyship, rather than saviorship, and thus center opportunities, experiences, and voices of individuals from minoritized groups; for more conceptual clarity and concrete examples of this distinction, see Williams et al. (2021).

Researchers also need to develop strategies to minimize the risks and burdens placed on individuals from minoritized groups who are often tasked with “helping” potential allies feel compassion or take compassionate action by exposing their own painful experiences. The costs of this emotional labor should not be incurred disproportionately by those whom allyship seeks to support.

## Conclusion

Compassion is central to social justice, insofar as social justice aims to reform discriminatory systems that enact systematic harms. Some argue that actions to promote social justice that are not based in compassion risk becoming performative or ineffectual, reinforcing low-effort behaviors like expressions of support on social media, rather than high-effort behaviors like protest actions to raise awareness about unfair policies or encourage legislative reforms (LeBlanc et al., 2022). Our findings suggest that cultivating compassion for others could promote a sense of allyship with communities of color that have experienced racism, irrespective of political ideology. This supports the importance of strategic implementation of high-quality compassion training to promote antiracist behavior which can ultimately lead to antiracist policy change. Importantly, allyship and political activism are known to be shaped by motivated reasoning. In recognizing the impact of emotional connection versus disengagement, the strategies and research directions we propose can promote compassionate responses to systemic suffering by anchoring recognition of racist policies and practices in greater awareness and attention to personal accounts of suffering and its impacts as experienced by minoritized individuals and groups. Importantly, future research will need to test whether increasing compassion goes beyond self-reports of enhanced allyship and leads to actual behavior that promotes greater justice, equity, and inclusion. It is also important to test the efficacy of compassion-focused strategies while centering communities of color in defining and assessing what constitutes effective allyship.

## Data availability statement

The raw data supporting the conclusions of this article will be made available by the authors, without undue reservation.

## Ethics statement

IRB approval for the study was obtained from the University of California, San Diego (Protocol #20042949). The patients/participants provided their written informed consent to participate in this study.

## Author contributions

MK, RR, and CB contributed to the conception and design of the study. MK and CB collected the data. MK performed the statistical analysis and wrote the first draft of the manuscript. RR and CB wrote sections of the manuscript. All authors contributed to the article and approved the submitted version.

## Funding

This work was supported by the Center for Empathy and Technology within the T. Denny Sanford Institute for Empathy and Compassion at the University of California San Diego. The use of REDCap for this work was supported by NIH/NCATS Colorado CTSA Grant number UL1 TR002535.

## Conflict of interest

The authors declare that the research was conducted in the absence of any commercial or financial relationships that could be construed as a potential conflict of interest.

## Publisher’s note

All claims expressed in this article are solely those of the authors and do not necessarily represent those of their affiliated organizations, or those of the publisher, the editors and the reviewers. Any product that may be evaluated in this article, or claim that may be made by its manufacturer, is not guaranteed or endorsed by the publisher.

## Author disclaimer

The paper’s contents are the authors’ sole responsibility and do not necessarily represent official NIH views.
